# Simulation on Pore Formation from Polymer Solution at Surface in Contact with Solid Substrate via Thermally Induced Phase Separation

**DOI:** 10.3390/membranes11070527

**Published:** 2021-07-13

**Authors:** Yasushi Mino, Naruki Fukukawa, Hideto Matsuyama

**Affiliations:** 1 Division of Applied Chemistry, Graduate School of Natural Science and Technology, Okayama University, 3-1-1 Tsushima-naka, Kita-ku, Okayama 700-8530, Japan; ymino@okayama-u.ac.jp; 2 Research Center for Membrane and Film Technology, Department of Chemical Science and Engineering, Kobe University, 1-1 Rokkodaicho, Nada-ku, Kobe 657-8501, Japan; 1314772t@stu.kobe-u.ac.jp

**Keywords:** polymeric membrane, thermally induced phase separation, surface porosity, phase field simulation, solid surface

## Abstract

The formation of porous structures from polymer solutions at the surface in contact with various solid surfaces via a thermally-induced phase separation (TIPS) process is investigated. The pore formation process at the bulk and the surface of the poly(methyl methacrylate)/cyclohexanol solution is simulated with a model based on the phase field method. When the compatibilities between the polymer-rich phase formed by the phase separation and the solid surface are high or low, surface porosity decreases. In contrast, for the solid surface having similar compatibilities with the polymer and solvent, high surface porosity is achieved. This indicates that the compatibility between the solid surface and polymer solution is important, and that optimal compatibility results in high surface porosity. The knowledge obtained in this work is useful to design the coagulation bath component in the membrane preparation process by TIPS for achieving high surface porosity.

## 1. Introduction

The thermally induced phase separation (TIPS) of a polymer solution is one of the major methods for preparing porous polymeric membranes [1,2,3]. In this process, a homogeneous solution of a polymer and a solvent at a high temperature is cast onto a substrate or spun in the form of a hollow fiber, and immersed in a cooling bath to induce phase separation and solidification. When the polymer solution is cooled down to a temperature below the spinodal decomposition temperature, the homogeneous solution starts to separate into two phases, that is, a polymer-rich phase and a solvent-rich phase (polymer-lean phase), which is a well-known spinodal decomposition mechanism [4]. After a certain period of phase separation, the polymer-rich phase is solidified as a membrane matrix by crystallization or glass transition, while the solvent-rich phase forms membrane pores. The resultant porous membrane can be used in various separation processes, including water treatment [5,6,7], battery separation [8,9], and membrane distillation [10,11], because of its diversified pore structure, well controlled porosity, and good mechanical property.

The surface pore structure is critical for determining the membrane performance [12,13,14,15] because the surface pore size and surface porosity are closely related to rejection and permeability. In the TIPS process, asymmetric membranes are mostly produced with a dense skin-layer at the surface resulting from solvent evaporation [5,16,17]. It is obvious that the presence of the dense skin-layer considerably increases the membrane water transport resistance and results in low water permeability. To avoid dense skin-layer formation in the TIPS process, diluents are added to the coagulation bath [16,18,19]. In such cases, porous structures were successfully formed on the membrane surfaces. This indicates the importance of phase separation at the surface. For phase transition [20,21] and phase separation of a polymer blend [22], the effects of the physicochemical surface properties of solid substrates have been experimentally investigated. These studies indicate the importance of the compatibility between the polymer solution and the solid substrate in phase separation.

Numerical simulation has been an effective approach for understanding the membrane formation mechanism via the TIPS process. Most studies have focused mainly on phase separation in bulk solutions [23,24,25,26]. A few studies explored the anisotropic morphological development near the surface when a superficial polymer concentration gradient [25,27,28] and a superficial temperature gradient [29,30,31] existed. However, the formation mechanism of the surface structure is not fully understood.

In this study, we investigated how the porous structure develops near the solid substrate during the TIPS of a polymer solution. The TIPS process was simulated using a numerical model based on the phase field method (PFM). The simulation model, which was used to study the formation mechanism of the pore structure at the midst of the membrane in our previous study [25], was developed to compute the phase separation of the polymer solution at the surface in contact with a solid surface having various compatibilities for the polymers and solvents. Based on the results, we propose a mechanism for the surface structure formation of polymer membranes via the TIPS process.

## 2. Numerical Simulation

### 2.1. TIPS Simulation

The TIPS of the polymer solution was simulated by PFM [24,32]. Phase-field variable *ϕ* is defined as the polymer volume fraction (vol/vol). Considering a polymer solution system with bulk free energy and gradient energy, the time evolution equation is described by the Cahn–Hilliard equation [32]:

(1) $$\frac{\partial \phi }{\partial t}=\nabla \cdot \left[M\left(\phi \right)\nabla \mu \right],$$

where *M*(*ϕ*) is the mobility depending on the composition and temperature, and *μ* is the chemical potential. The chemical potential is expressed as the functional derivative of the free energy functional, *F*:

(2) $$\mu =\frac{\delta F}{\delta \phi }=\frac{\partial f\left(\phi \right)}{\partial \phi }-2\kappa \nabla^{2}\phi ,$$

where *f* is the free energy of polymer–solvent mixing, and *κ* is the gradient energy parameter.

According to the Flory–Huggins theory [33], *f* is written as

(3) $$f=RT\left[\frac{\phi \ln\phi }{N_{p}}+\left(1-\phi \right)\ln\left(1-\phi \right)+\chi \phi \left(1-\phi \right)\right],$$

where *N*_p_ is the degree of polymerization and *χ* is the polymer–solvent interaction parameter. Parameter *χ* determines the enthalpy contribution toward mixing and is usually expressed as a function of temperature *T*. The gradient energy parameter *κ* is estimated from Debye’s approximation [34]:

(4) $$\kappa =RT\chi R_{G}{}^{2}/6,$$

where *R*_G_ is the radius of gyration of polymer. The mobility *M* is calculated as

(5) $$M=\frac{\phi \left(1-\phi \right)}{\alpha {\left(1-\phi \right)}^{2}+\phi \left(3-2\phi \right)}\frac{D_{1}}{RT},$$

where *D*_1_ is the solvent self-diffusion coefficient, *α* is the ratio of *D*_1_ to *N*_p_*D*_2_, where *D*_2_ is the polymer self-diffusion coefficient. Further details of this model, including the determination method of each parameter, are given in Refs. [24,35,36].

### 2.2. Boundary Conditions for Solid Surfaces with Various Compatibilities for Polymers and Solvents

**Figure 1:**
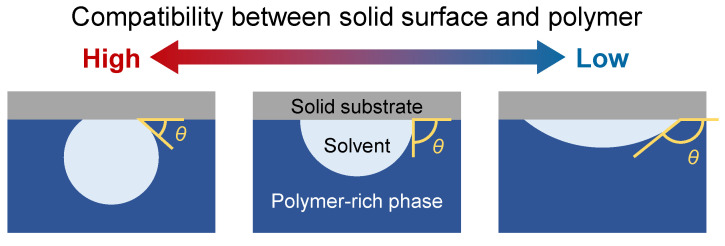
Schematic of contact angle representing compatibility between solid surface and polymer.

In our previous study [25], a periodic boundary condition was reasonably applied to all directions because we focused on the phenomena in the bulk of the membrane. However, to investigate the mechanism for structure formation at the surface in contact with the solid substrate, the boundary condition for the solid surface with various compatibilities for the polymer and solvent was required. Here, the compatibility was represented by contact angle *θ* of the polymer-rich phase in the solvent-rich phase formed by phase separation on the solid surface; the solid surface with *θ* < 90° or *θ* > 90° has a high or low compatibility with the polymer, respectively. The schematic of the contact angle is shown in Figure 1.

The contact angle *θ* was described by a simple geometric model [37]. The normal-direction derivations of *ϕ* to the solid surface are given by

(6) $$\frac{\nabla \phi }{\left|\nabla \phi \right|}\cdot n_{\mathrm{solid}}=\cos\theta ,$$

where **n**_solid_ is the unit normal vector to the solid surface. In this study, because we set the solid surface for the bottom- and top-side boundaries, **n**_solid_ = (0, 0, ±1) gives

(7) $$\pm \frac{\partial \phi }{\partial z}=\sqrt{{\left(\frac{\partial \phi }{\partial x}\right)}^{2}+{\left(\frac{\partial \phi }{\partial y}\right)}^{2}+{\left(\frac{\partial \phi }{\partial z}\right)}^{2}}\cos\theta .$$

Consequently, the boundary conditions of *ϕ* for the bottom and top walls can be expressed as

(8) $$\frac{\partial \phi }{\partial z}=\pm \frac{\cos\theta }{\sqrt{1-\cos^{2}\theta }}\sqrt{{\left(\frac{\partial \phi }{\partial x}\right)}^{2}+{\left(\frac{\partial \phi }{\partial y}\right)}^{2}}.$$

Furthermore, because the materials do not transfer through the solid surface, the normal-direction derivation of chemical potentials *μ* to the solid surface is given by

(9) $$\nabla \mu \cdot n_{\mathrm{solid}}=0.$$

Consequently, the boundary conditions for *μ* at the top and bottom walls can be expressed as

(10) $$\frac{\partial \mu }{\partial z}=0.$$

### 2.3. Simulaation Condition

We considered the poly(methyl methacrylate) (PMMA)/cyclohexanol system, which is a typical polymer solution that exhibits liquid/liquid phase separation. Note that the present simulation model can be generally applied to other systems with appropriate parameters. The parameters reported in Refs. [24,25] were used in this study. The degree of polymerization of PMMA was *N*_p_ = 150. The temperature-dependent parameter *χ* was given by [24]

(11) χ=−5.068+1900.6/T(K).

**Figure 2:**
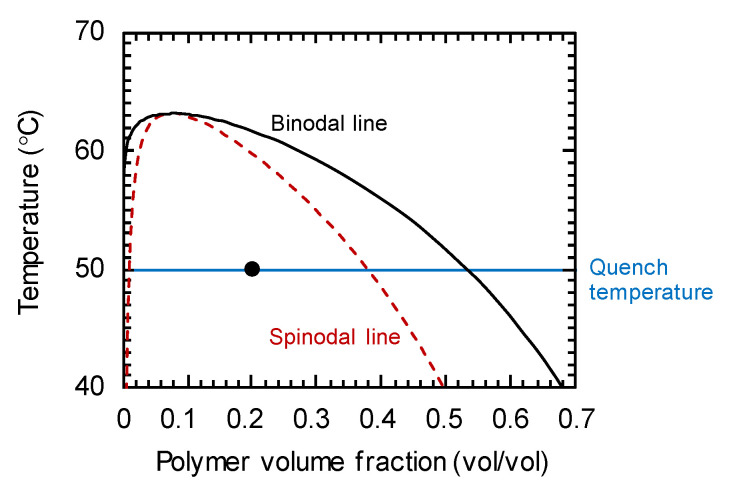
Phase diagram of PMMA/cyclohexanol system. The solid and dashed lines represent the binodal and spinodal lines, respectively. The condition targeted in this study is plotted in this diagram.

The phase diagram of the PMMA/cyclohexanol system was shown in Figure 2. The solid and dashed lines represent the binodal and spinodal lines, respectively. The phase diagram has the critical point very close to the pure solvent axis, which is a characteristic of polymer solution system. In this study, the initial polymer concentration and quench temperature were *ϕ*_0_ = 0.20 and *T* = 50 °C, respectively, which is plotted in this diagram. In this system, the solvent-rich phase can be considered as a pure solvent because of its considerably low polymer concentration. The variations in mobility *M* are provided in Ref. [24]. At the fixed quench temperature of *T* = 50 °C, *M* was a function of only polymer concentration, and *M* exhibited a maximum of 1.52 × 10^-10^ (cm^2^ mol)/(J s) at *ϕ* ≈ 0.07 and decreased to 0 toward the pure component limits (Figure 1 in Ref. [24]).

The Cahn–Hilliard equation can be scaled using the following dimensionless quantities [24]:

(12) $$x^{*}=x/L_{0},t^{*}=\left(2\kappa M_{0}/L_{0}{}^{4}\right)t,f^{*}=f/RT,M^{*}=M/M_{0},\nabla^{*}=L_{0}\nabla$$

where *M*_0_ is a scaling constant having units of mobility, and *L*_0_ is the scaling length given by

(13) $$L_{0}=a{\left(2\kappa /RT\right)}^{1/2},$$

where *a* is an adjustable parameter, which was set to 1 in this study. Thus, Equations (1) and (2) become

(14) $$\frac{\partial \phi }{\partial t^{*}}=\nabla^{*}\cdot \left[M^{*}\left(\phi \right)\nabla^{*}\left(a^{2}\frac{\partial f^{*}\left(\phi \right)}{\partial \phi }-\nabla^{*}{}^{2}\phi \right)\right].$$

This dimensionless equation was solved using a finite difference scheme for both time and spatial discretization.

**Figure 3:**
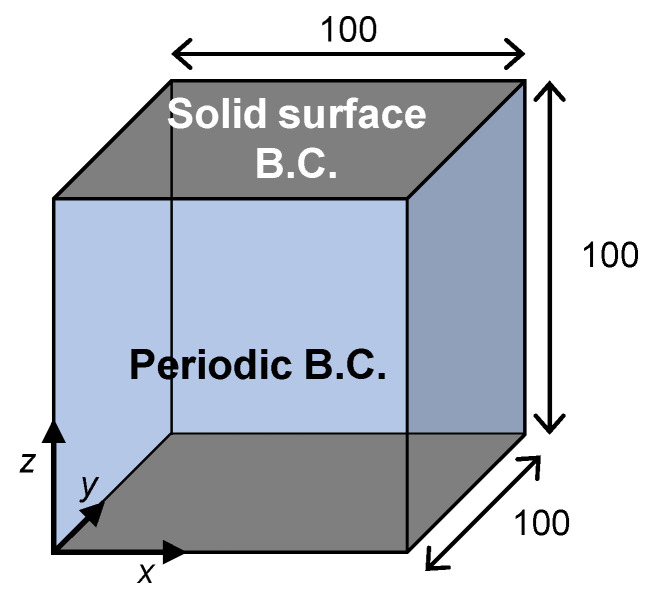
Computational system used to simulate thermally induced phase separation (TIPS) of a polymer solution with a solid surface boundary condition (B.C. in the figure) for the top and bottom boundaries and periodic boundary conditions for the side boundaries.

The calculations were conducted using an original program, the validity of which was demonstrated in our previous work [25]. The computational system for the TIPS simulations is illustrated in Figure 3. The dimensionless size of computational domain was L_x_^*^ × L_y_^*^ × L_z_^*^ = 100 × 100 × 100. The solid surface boundary condition, which was described in Section 2.2, was applied to the top and bottom boundaries, while the periodic boundary condition was applied to the side boundaries. The initial polymer concentration was set to *ϕ*(**x**) = *ϕ*_0_ + δ*ϕ*(**x**), where δ*ϕ*(**x**) represents an infinitesimal fluctuation of polymer concentration and was provided by a random number generation algorithm.

## 3. Results and Discussion

### 3.1. Evolution of Membrane Morphology in Bulk of Membrane

**Figure 4:**
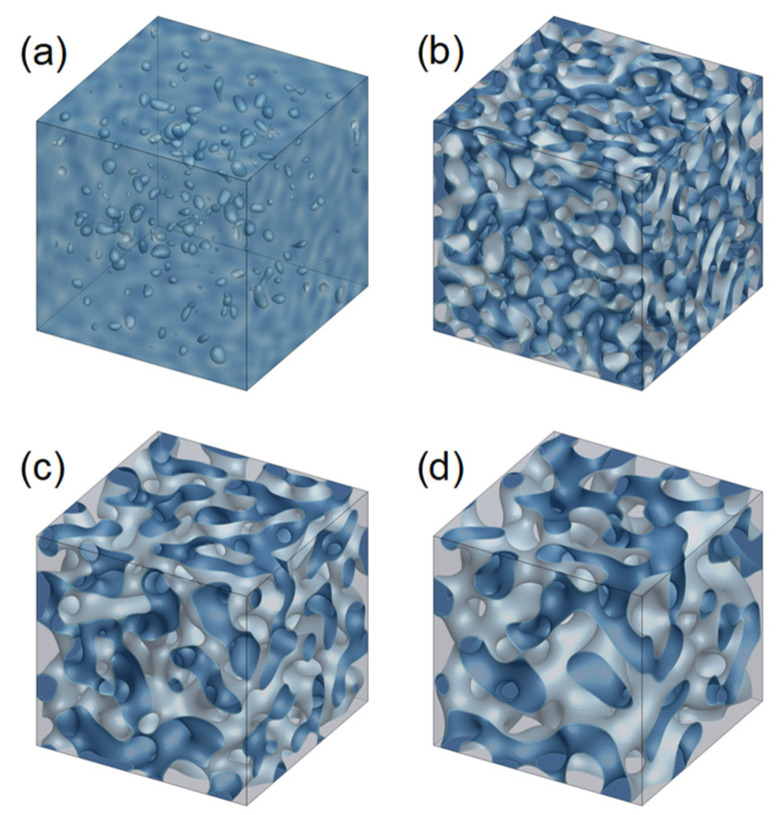
Snapshots depicting the evolution of pore morphology at the inside part of the membrane, which was obtained with an initial polymer concentration of *ϕ*_0_ = 0.20, by applying the periodic boundary condition in all directions. Snapshots were obtained at t^*^ = (**a**) 0.02, (**b**) 0.04, (**c**) 0.30, and (**d**) 0.80. The isosurfaces of *ϕ* = 0.15 are depicted. The blue- and gray-colored areas represent the polymer-rich and solvent-rich phases, respectively.

First, the evolution of membrane morphology in the bulk of the membrane, which was obtained by applying the periodic boundary condition in all directions, was calculated (Figure 4). Hereafter, in the snapshots, the isosurfaces of *ϕ* = 0.15 are depicted, and the blue- and gray-colored areas represent the polymer-rich and solvent-rich phases, respectively. The initial minute fluctuations in the polymer concentration rapidly generated a solvent-rich phase (Figure 4a). As the phase separation progressed, the solvent-rich phases grew and connected with each other (Figure 4b,c), resulting in the formation of bicontinuous morphology (Figure 4d). This tendency of morphological development is in good agreement with that reported in the previous results, which were obtained with an initial polymer concentration of *ϕ*_0_ = 0.30 [25].

### 3.2. Evolution of Membrane Morphology at Surface of Membrane

**Figure 5:**
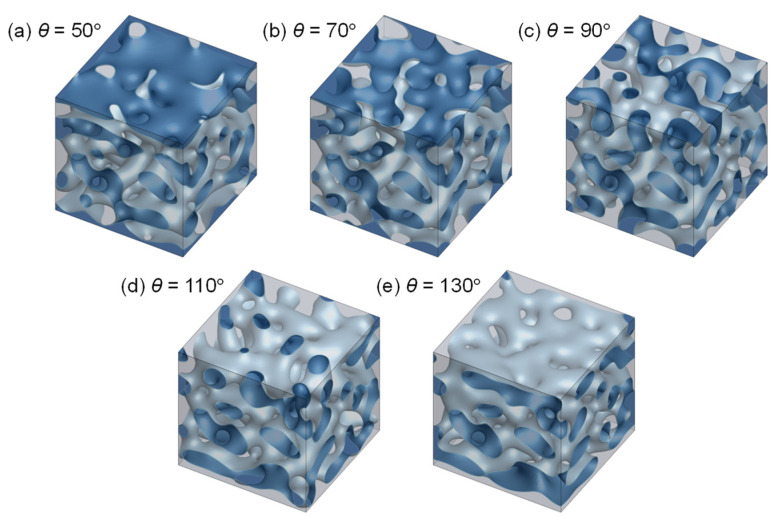
Effect of contact angle *θ* on the membrane structure. *θ* = (**a**) 50°, (**b**) 70°, (**c**) 90°, (**d**) 110°, and (**e**) 130°. t^*^ = 0.8.

Figure 5 shows typical membrane morphologies (t^*^ = 0.8) calculated using the solid surface boundary condition. The effect of the solid surface contact angle *θ* = 50°, 70°, 90°, 110°, and 130° on the membrane structure was investigated. All membranes showed almost the same structures in the bulk, which were similar to those formed in the system where the periodic boundary condition was applied in all directions (Figure 4d). However, the surface structures drastically varied according to *θ*, associated with the compatibility between the polymer and the solid surface. At the membrane surface, the fraction of the solvent-rich phase increased with the decrease in the compatibility between the polymer and the solid surface, while the polymer-rich phase fraction increased with an increase in this compatibility. In the case of high (*θ* = 50°, Figure 5a) or low (*θ* = 130°, Figure 5e) compatibility between the polymer and the solid surface, the polymer-rich (blue) or solvent-rich (gray) phase occupied the large part of the membrane surface. When the solid substrate had similar compatibilities with the polymer-rich phase and the solvent-rich phase (*θ* = 90°, Figure 5c), the surface structure became similar to the bulk structure.

**Figure 6:**
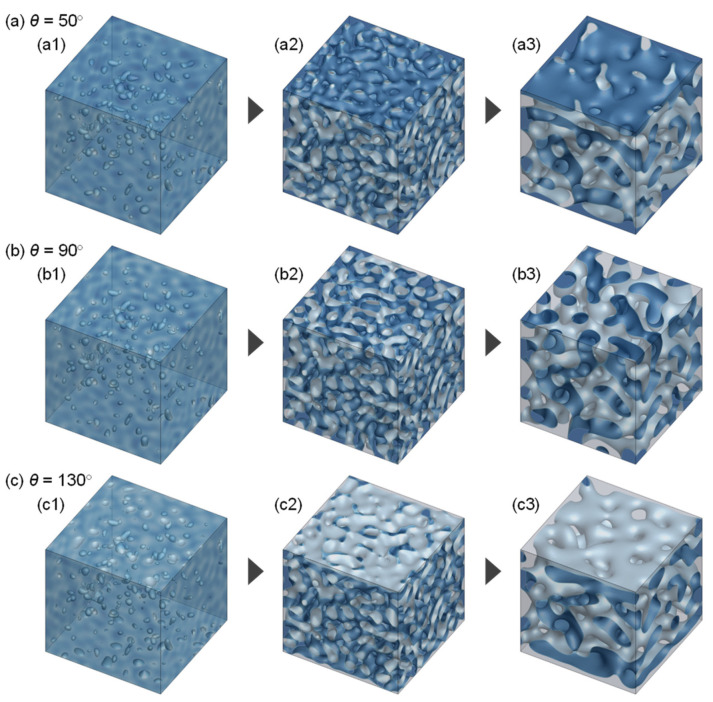
Growth of surface morphology. *θ* = (**a**) 50°, (**b**) 90°, and (**c**) 130°. The times when the snapshots were obtained were t^*^ = (**1**) 0.02, (**2**) 0.04, and (**3**) 0.5 for all the cases.

The time-course of morphology growth is shown in Figure 6 for *θ* = 50°, 90°, and 130°. In all cases, the structure growth in the bulk was similar to that described in Figure 4. The surface structure development, however, varied with *θ*. For *θ* = 90° (Figure 6b), the surface morphology grew in the same way as that in the bulk. Thus, the surface structure was almost identical to that of the bulk structure. At the higher compatibility between the polymer and the solid surface (*θ* = 50°), the membrane surface was almost covered by the polymer-rich phase after a short time (Figure 6(a2)), and the polymer-rich phase at the surface grew (Figure 6(a3)). On the other hand, at the lower compatibility between the polymer and the solid surface (*θ* = 130°), the solvent-rich phase concentrated quickly near the surface (Figure 6(c2)), and most of the surface area was covered by the solvent-rich phase (Figure 6(c3)).

**Figure 7:**
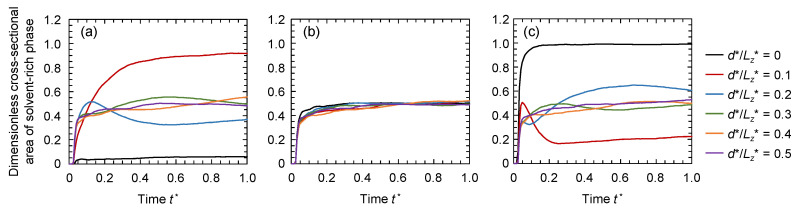
Time variations of the dimensionless cross-sectional area of the solvent-rich phase at different depths from the solid surface, d^*^/L_z_^*^ = 0, 0.1, 0.2, 0.3, 0.4, and 0.5, for *θ* = (**a**) 50°, (**b**) 90°, and (**c**) 130°.

Figure 7 shows the time variations of the dimensionless cross-sectional area of the solvent-rich phase at different depths from the solid surface, d^*^/L_z_^*^ = 0, 0.1, 0.2, 0.3, 0.4, and 0.5, where L_z_^*^ is the height of the computational domain. Note that the lines of *d* = 0 represent the surface area of the solvent-rich phase, while those of d^*^/L_z_^*^ = 0.5 represent the area at the center of the polymer solution. When the solid substrate had similar compatibilities with the polymer-rich phase and solvent-rich phase (*θ* = 90°, Figure 7b), the cross-sectional area of the solvent-rich phase rarely varied at any depth position, and the cross-sectional area was almost the same even at the surface (d^*^/L_z_^*^ = 0). At *θ* = 50° (Figure 7a), the surface area of the solvent-rich phase (surface porosity) was considerably low because of the compatible polymer contact with the solid surface. The cross-sectional area just below the surface (d^*^/L_z_^*^ = 0.1) increased because the polymer moved to the solid surface, while the polymer concentration just below the surface decreased. In contrast, at *θ* = 130° (Figure 7c), the surface area of the solvent-rich phase was largest, almost equal to 1.0, suggesting full coverage of the solvent-rich phase. The cross-sectional area just below the surface (d^*^/L_z_^*^ = 0.1) decreased due to the decrease in solvent concentration at this position.

The fully covered solvent-rich phase at the surface is swept away by the subsequent solvent exchange in the real membrane preparation process. Thus, the surface porosity of the membrane in this case was dependent on the cross-sectional area of the solvent-rich phase just below the surface. At *θ* = 50° and 130°, the cross-sectional areas for d^*^/L_z_^*^ = 0.3–0.5 did not considerably vary and were close to those at *θ* = 90°. This suggests that the bulk structures were similar even at *θ* = 50° and 130°.

**Figure 8:**
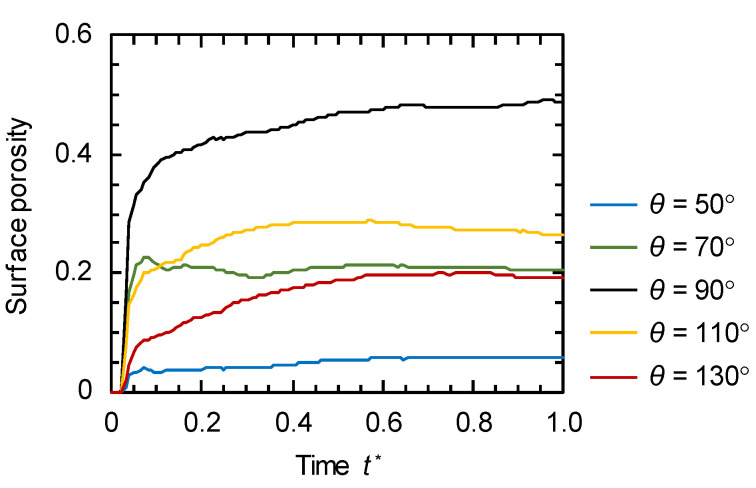
Time variations of surface porosity at *θ* = 50°, 70°, 90°, 110°, and 130°.

Generally, the dimensionless cross-sectional area of the solvent-rich phase at the surface corresponds to the surface porosity of the membrane. However, as described above, the cross-sectional area of the solvent-rich phase just below the surface should be considered as the surface porosity at high *θ.* Here, the dimensionless cross-sectional areas at the surface were considered as the surface porosity at *θ* = 50°, 70°, and 90°. At *θ* = 110° and 130°, the smallest cross-sectional areas along the *z* direction were considered as the surface porosity. The time variations of the surface porosities at various *θ* are shown in Figure 8. When the solid surface was highly compatible with the polymer (*θ* = 50°), the surface porosity was low because the polymer was concentrated at the surface. On the other hand, in the case of the low compatibility between the solid surface and the polymer (*θ* = 130°), the surface porosity was also low because the solvent was concentrated at the surface and the amount of solvent-rich phase just below the surface decreased (Figure 7c). Consequently, when the solid substrate had similar compatibilities with the polymer-rich phase and solvent-rich phase (*θ* = 90°), the highest surface porosity was achieved.

**Figure 9:**
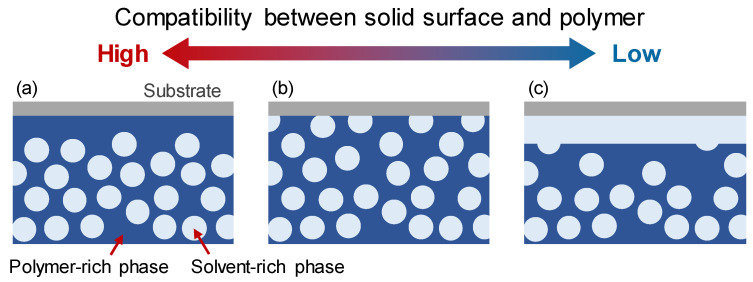
Illustration of surface structure development in cases of solid surfaces with (**a**) high, (**b**) neutral, and (**c**) low compatibility with polymer.

Based on the numerical results, we propose the mechanism of surface structure development, which is schematically illustrated in Figure 9. When the compatibility between the solid surface and the polymer is high, the polymer-rich phase is concentrated at the interface. Thus, the pores generated at the beginning of phase separation disappear at the surface with the progress of phase separation (Figure 6a). When the solid surface has similar compatibilities with the polymer and solvent, both the polymer and solvent are not particularly attracted to the solid surface, and the phase separation progresses in a manner similar to that inside the membrane. The pore size increases while maintaining a constant solvent-rich phase fraction at the surface (Figure 7b). When the solid surface has high compatibility with the solvent, the solvent moves to the interface; thus, the polymer-rich phase is concentrated just below the surface. As a result, the surface porosity decreases (Figure 7c) after the top layer fully covered by the solvent-rich phase is swept away. Thus, the surface porosity is highly affected by the solid surface property, and the optimal solid surface property exists to achieve high surface porosity.

## 4. Conclusions

We simulated the formation of porous structures from the polymer solution in contact with the solid surface via the TIPS process. The simulation model was based on PFM and developed to describe the phase separation of the polymer solution in contact with the solid substrates with various compatibilities with the polymer. When the polymer solution was in contact with the solid surface having high compatibility with the polymer, the polymer was concentrated near the surface, resulting in a decrease in surface porosity. On the other hand, the contact of the solid surface having high compatibility with the solvent resulted in the formation of a solvent-rich phase layer at the surface. The surface porosity of the membrane in this case was considered to be the cross-sectional area of the solvent-rich phase just below the surface due to the sweeping away of the surface solvent layer. Because the solvent moved to the interface, and thus the polymer-rich phase was concentrated just below the surface, the surface porosity also decreased in this case. When the solid surface had similar compatibilities with the polymer and solvent, both the polymer and solvent were not particularly attracted to the solid surface, and the highest surface porosity was achieved. Thus, the surface porosity was highly affected by the solid surface property, and the optimal solid surface property existed to achieve high surface porosity.

In this study, the phase separation at the interface between the polymer solution and the solid surface was investigated. The results obtained in this work can be applied to the selection of the coagulation bath component in the general membrane preparation process via TIPS. The compatibility of the coagulation bath component with the polymer and solvent is very important for controlling the membrane surface structure, and the selection of the bath component with moderate compatibility with both the polymer and solvent may be useful to achieve high surface porosity. Although the polymer/solvent systems which can be applied for TIPS method are limited, the coagulation bath can be prepared with a multicomponent mixture to tune the compatibility.
